# A rare homozygous missense *GDF2* (BMP9) mutation causing PAH in siblings: Does BMP10 status contribute?

**DOI:** 10.1002/ajmg.a.62996

**Published:** 2022-10-19

**Authors:** Paul Upton, Susan Richards, Angela Bates, Karen Y. Niederhoffer, Nicholas W. Morrell, Susan Christian

**Affiliations:** ^1^ Department of Medicine, Heart and Lung Research Institute University of Cambridge Cambridge UK; ^2^ Pediatric Pulmonary Hypertension Service Stollery Children's Hospital Edmonton Alberta Canada; ^3^ Department of Pediatrics University of Alberta Edmonton Alberta Canada; ^4^ Department of Medical Genetics University of Alberta Hospital Edmonton Alberta Canada

**Keywords:** BMP, bone morphogenetic protein, GDF, pulmonary arterial hypertension

## Abstract

Pulmonary arterial hypertension (PAH) is a disease characterized by pathological remodeling of the pulmonary vasculature causing elevated pulmonary artery pressures and ultimately, right ventricular failure from chronic pressure overload. Heterozygous pathogenic *GDF2* (encoding bone morphogenetic protein 9 (BMP9)) variants account for some (>1%) adult PAH cases. Only three pediatric PAH cases, harboring homozygous or compound heterozygous variants, are reported to date. Ultra‐rare pathogenic *GDF2* variants are reported in hereditary hemorrhagic telangiectasia and overlapping disorders characterized by telangiectasias and arteriovenous malformations (AVMs). Here, we present two siblings with PAH homozygous for a *GDF2* mutation that impairs BMP9 proprotein processing and reduces growth factor domain availability. We confirm an absence of measurable plasma BMP9 whereas BMP10 levels are detectable and serum‐dependent endothelial BMP activity is evident. This contrasts with the absence of activity which we reported in two children with homozygous pathogenic *GDF2* nonsense variants, one with PAH and one with pulmonary AVMs, both with telangiectasias, suggesting loss of BMP10 and endothelial BMP activity in the latter may precipitate telangiectasia development. An absence of phenotype in related heterozygous *GDF2* variant carriers suggests incomplete penetrance in PAH and AVM‐related diseases, indicating that additional somatic and/or genetic modifiers may be necessary for disease precipitation.

## 1 INTRODUCTION

Pulmonary arterial hypertension (PAH) is a disease, characterized by progressive vasculopathy of the pulmonary arteries, which carries a high level of morbidity and mortality (Abman et al., 2015). Pediatric PAH is defined as a mean pulmonary artery pressure (mPAP) ≥20 mmHg after 3 months of age with a pulmonary capillary wedge pressure (PCWP) or left ventricular end diastolic pressure (LVEDP) of <15 mmHg and pulmonary vascular resistance indexed to body surface area (PVRI) ≥3 Wood Units.m^2^ (WU.m^2^) (Rosenzweig et al., 2019). Unlike adult PAH, the majority of pediatric PAH cases are categorized as idiopathic PAH (IPAH), heritable PAH (HPAH), or PAH associated with congenital heart disease (PAH‐CHD) (Abman et al., 2015). The proportionate contributions of known PAH‐causing gene variants differs between adults and children (Welch & Chung, 2020). For example, pathogenic *BMPR2* variants explain 11%–40% of IPAH (Koehler et al., 2004; Morisaki et al., 2004) and over 75% HPAH (Machado et al., 2015; Soubrier et al., 2013) cases in adults, whilst accounting for 10%–16% (Harrison et al., 2005; Rosenzweig et al., 2008; van Loon et al., 2009) of pediatric PAH cases. Conversely, pathogenic *TBX4* (Kerstjens‐Frederikse et al., 2013; Levy et al., 2016) and ACVRL1 (Levy et al., 2016) variants are proportionately higher in pediatric PAH than adult cases. For *GDF2*, the known genetic pediatric PAH cases to date constitute one homozygous missense variant carrier and a compound heterozygote with a missense variant on one allele and a large deletion encompassing *GDF2* and *BMP10* on the second chromosome (Gallego et al., 2021; Wang et al., 2016).

## 2 CLINICAL DESCRIPTION

**FIGURE 1:**
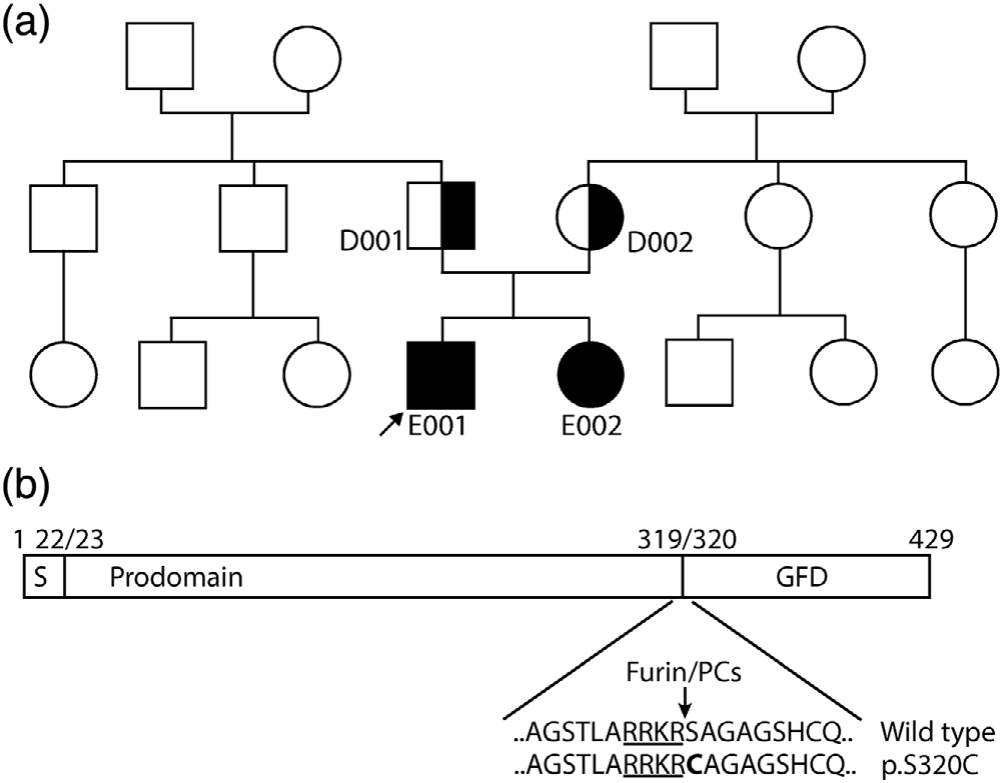
Family pedigree for the p.S320C variant and impact on BMP9 processing. (a) Family pedigree showing the inheritance of the pathogenic *GDF2* variant resulting in homozygous p.S320C BMP9 variants in the proband (E001, indicated by arrow) and younger sibling (E002), both diagnosed with PAH. The unaffected heterozygous parents (D001 and D002) are also shown. (b) Representation of the primary structure of the BMP9 preproprotein encoded by the GDF2 gene. (S = secretion signal peptide, GFD = growth factor domain). The amino acid sequence of the furin/prohormone convertase (PC) cleavage site between amino acids 319 and 320 is indicated by an arrow and the consensus motif is underlined. The deleterious missense variant introducing a Cysteine residue (**C**) is in bold.

The pedigree for the family is shown in Figure 1a and a detailed clinical description is provided in the Supplement. The proband (E001) was diagnosed with IPAH at 3 months of age and remained stable on therapy up to the time of blood sampling at 11 years of age. He is classified as WHO class 2 as he experiences shortness of breath with exercise (Lammers et al., 2011). At 11 years of age, heart rate was 93 bpm and systemic blood pressure was 105/60. His 6‐minute walk distance was 475 m (51% predicted for age), with good heart rate and blood pressure variability and an oxygen saturation drop from 95% to 91%. On recent cardiac catheterization, his mPAP was 33 mmHg, transpulmonary gradient (TPG) was 24 mmHg, cardiac index was 3.56 L/min/m^2^ and PCWP was 9 mmHg with a PVRI of 6.9 WU.m^2^. He does not have reactivity with acute vasoreactivity testing.

The sister of the proband (E002) was diagnosed with IPAH aged 4 years and was aged 8 years at the time of blood sampling. On cardiac catheterization aged 5 years, mPAP was 27 mmHg, TPG was 21 mmHg, PCWP was 6 and PVRI was 6.23 WU.m^2^, with a normal cardiac index (3.38 L/min/m^2^). Her WHO functional classification is 1–2 as she intermittently experiences shortness of breath upon exercise. Upon examination aged 8, heart rate was 81 bpm and systemic blood pressure was 117/54. Her 6‐minute walk distance was 460 m, with good heart rate and blood pressure variability. Her saturation declined from 94% to 93% and post‐test Borg dyspnea score was 5.

Neither parent (father = 41 years; mother = 38 years) exhibited any health concerns. Echocardiographic assessment indicated the right and left ventricles were normal with ejection fractions >55%, tricuspid annular plane systolic excursion 2.1–2.2 mm and normal RVSPs (father = 23 mmHg; mother = 17 mmHg). There is no recorded history of epistaxis, telangiectasias, or arteriovenous malformations (AVMs) in this family.

## 3 METHODS

### 3.1 Editorial policies and ethical considerations

The collection of patient samples was approved by the University of Alberta Research Ethics Office (Pro00098029) and sampling from controls (*n* = 5 females, *n* = 7 male) approved by the Cambridge East National Research Ethics Service Committee (REC Reference 11/EE/0297). Written informed consent was obtained for all participants.

### 3.2 Sequencing

Blood samples were collected and submitted to Prevention Genetics (Marshfield, WI) for sequencing through a 14 gene PAH panel which included the following genes: *ACVRL1*, *AQP1*, *BMPR1B*, *BMPR2*, *CAV1*, *EIF2AK4*, *ENG*, *GDF2*, *KCNA5*, *KCNK3*, *SMAD4*, *SMAD9*, *SOX17*, and *TBX4*.

### 3.3 BMP plasma concentrations and serum‐derived activity

For ELISA, EDTA plasma samples were collected and aliquoted prior to freezing. Circulating BMP9 and pBMP10 levels were measured as previously published (Hodgson et al., 2020). BMP10 growth factor domain (GFD) levels were measured using the BMP10 Duoset® ELISA (R&D Systems, Minneapolis, MN) with BioRad Assay buffer A (Hercules, CA) containing 0.2% (v/v) heat‐inactivated goat serum (Abcam, Cambridge, UK), 0.5% (v/v) Triton x‐100 and 4.5 mM EDTA to avoid matrix effects and improve recovery.

For activity assays, serum samples were collected and aliquoted prior to freezing. Serum endothelial BMP activity was assayed using the HMEC1‐BRE luciferase reporter cell line for samples serially diluted in serum‐free medium to 2.5%, 5%, 7.5% and 10% (v/v) (Hodgson et al., 2020).

### 3.4 Statistical analysis

Statistical analysis of the HMEC1‐BRE serum activity data was by a one‐way ANOVA for each of the serum dilution factors tested. For each control sample the mean of the values from three experimental repeats was calculated for each dilution. For each test subject, the three values from the experimental repeats were compared to the means for the sex matched controls at the same serum dilution.

## 4 RESULTS

Sequencing of the children with PAH identified a homozygous missense variant in *GDF2* (c.958A > T), encoding BMP9. Both parents were heterozygous for this variant (Figure 1a). The parents are not consanguineous but are from the same community. This missense variant leads to a substitution of Serine at position 320 for a Cysteine (p.S320C) (Hodgson et al., 2020). Serine 320 occupies the furin cleavage site between the BMP9 prodomain and growth factor domain regions (Figure 1b). We previously reported an adult PAH patient with a heterozygous BMP9 p.S320C variant and expressed this protein, confirming this amino acid substitution leads to impaired processing of the BMP9 proprotein (Hodgson et al., 2020).

We questioned whether circulating BMP9 levels are altered in the affected patients or their parents. BMP9 was not detectable in either of the affected children. Reflecting our previous data, BMP9 levels were higher in female controls compared to male controls (Hodgson et al., 2020, 2021). BMP9 levels were low in both unaffected heterozygous parents relative to their sex‐matched controls.

**FIGURE 2:**
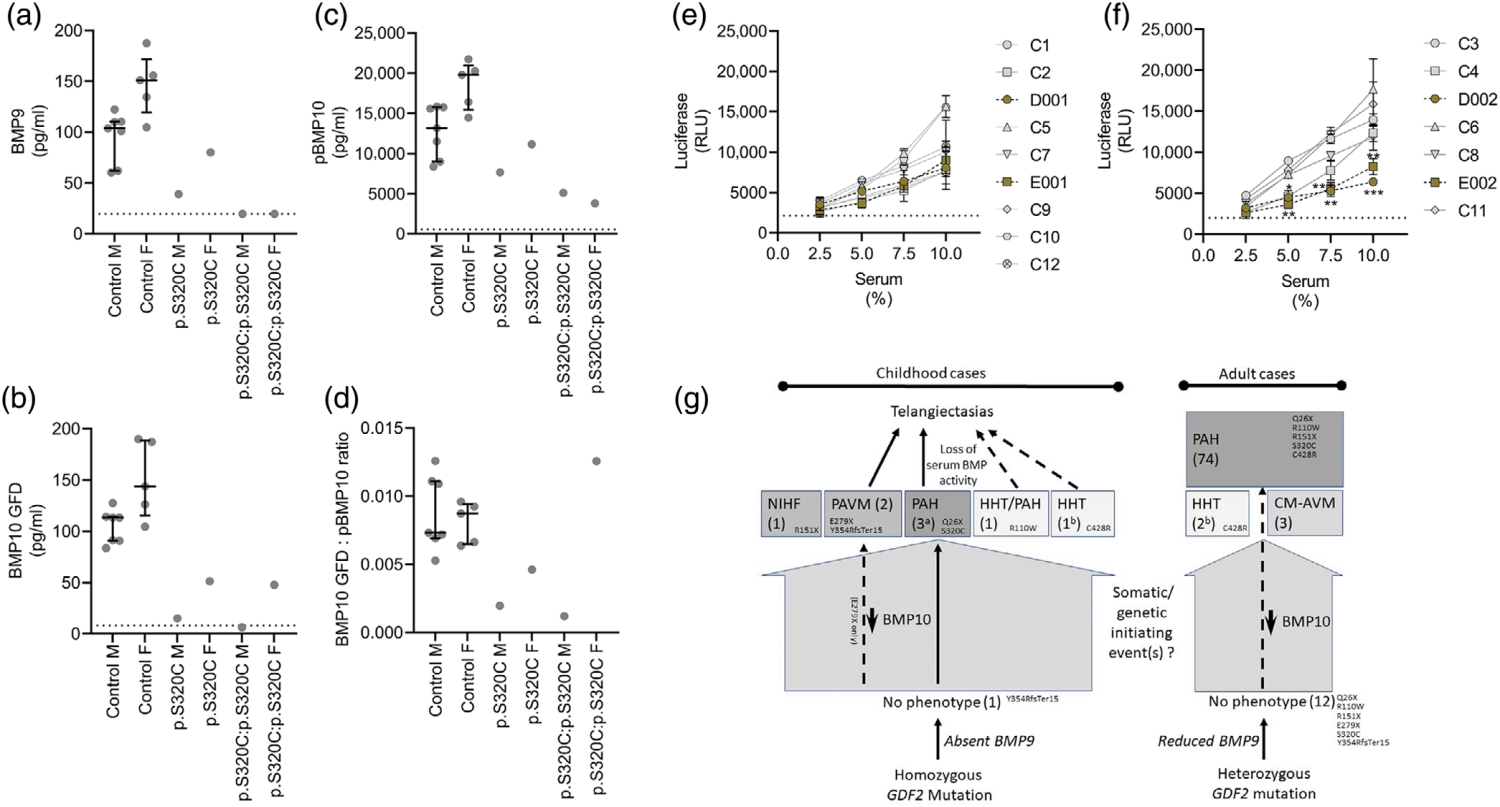
BMP9 and BMP10 levels and serum‐dependent BRE‐luciferase activity in p.S320C homozygotes and heterozygotes support a model of reduced penetrance. (a–d) ELISA for (a) BMP9, (b) pBMP10 and (c) BMP10 GFD in controls, p.S320C homozygous pediatric PAH patients and their unaffected p.S320C heterozygous parents. (d) Ratio of the BMP10 GFD and pBMP10 levels. (e, f) Serum‐derived BMP activity in samples from the same individuals as above for (E) males and (F) females. Activity data are mean ± SEM for experimental repeats performed on three separate occasions (two technical replicates per experiment). **p < 0.01; ***p < 0.005 relative to means of sex‐matched controls by one‐way ANOVA. (G) Reports of unaffected individuals with heterozygous or homozygous pathogenic *GDF2* variants (Table S1) suggests disease initiation may involve additional genetic and/or somatic events (gray arrows). Reduction of BMP10 levels measured in all (*solid black arrows*) or some (*dashed black arrows*) cases may contribute. Loss of serum derived BMP9/10 activity may lead to the emergence of telangiectasias/AVMs. Numbers in parentheses are numbers of reported individuals with pathogenic variants (^a^ = siblings, ^b^ = from the same pedigree). Exemplar variants are shown (Q26X, R110W, R151X, E279X, S320C, Y354RfsTer15, C428R). CM‐AVM, cutaneous malformation‐arteriovenous malformation; HHT, hereditary hemorrhagic telangiectasia; NIHF, non‐immune hydrops fetalis; PAH, pulmonary arterial hypertension; PAVM, pulmonary arteriovenous malformation.

We previously reported that plasma BMP9 and BMP10 levels strongly correlate (Hodgson et al., 2020) and it is reported that BMP9 and BMP10 may circulate as heterodimers (Hodgson et al., 2020; Tillet et al., 2018). We therefore measured plasma BMP10 GFD levels (either free GFD or in a non‐covalent complex with furin‐cleaved prodomain) and pBMP10 (either uncleaved or furin‐cleaved prodomain‐associated BMP10). Interestingly, BMP10 GFD levels were just detectable in the unaffected father (15.10 pg/ml), but below the detection limit in the male child (6.21 pg/ml) with PAH (Figure 2b). In contrast, BMP10 GFD levels were low, but detectable and similar in the mother and female PAH child (51.43 and 47.94 pg/ml respectively). pBMP10 levels in the unaffected parents (father: 7686 pg/ml and mother: 11148 pg/ml) were lower than the sex matched controls (Figure 2c). pBMP10 levels were lower, but detectable in both children (Male child = 5126 pg/ml and female child = 3815 pg/ml). The BMP10 GFD:pBMP10 ratio (Figure 2d) was higher in the female child than the other family members, suggesting BMP10 processing and thus, the availability of the GFD may be increased.

BMP9 and BMP10 in the serum or plasma of individuals are responsible for the majority of endothelial BMP signaling activity (David et al., 2008; Hodgson et al., 2020; Laux et al., 2013), though plasma can clot so assaying serum is preferable. We compared serum activity for the patients and parents to sera from the same controls as the plasma samples assayed by ELISA. Serum‐derived endothelial BMP activity was still evident in the male parent and affected son, though toward the lower level of activity observed in the male controls, (Figure 2e). This contrasted with the mother and female PAH patient (Figure 2f), both of whom exhibited significantly lower serum‐dependent activity than the female controls.

## 5 DISCUSSION

We identified two siblings, both with PAH, with a homozygous missense variant in the *GDF2* gene. We demonstrate this leads to an absence of measurable plasma BMP9 in both children, while their unaffected heterozygous parents exhibit reduced levels compared to sex‐matched controls. This variant, p.S320C, previously identified as a heterozygous variant in adult PAH patients, impairs processing in vitro by disrupting the furin cleavage site between the BMP9 prodomain and GFD (Hodgson et al., 2020; Wang et al., 2019).

We recently presented the first plasma BMP9 measurements in two children with homozygous *GDF2* nonsense variants, one child with PAH and the other with diffuse pulmonary artery venous malformations, both with atypical facial telangiectasias (Hodgson et al., 2021). Here, we add to this rare genetic cohort, showing that 2 pediatric siblings with homozygous p.S320C missense variants have PAH and an absence of measurable BMP9. This is not due to reduced ELISA cross‐reactivity, as we previously reported that the expressed p.S320C protein is detected with equal efficiency to the wild type (Hodgson et al., 2020). We argue that evidence of pathogenicity is essential for assessing *GDF2* mutations, as in silico predictions of pathogenicity do not always translate to a functional defect (Hodgson et al., 2021).

In our previous study, both children with nonsense *GDF2* variants also exhibited an absence of pBMP10 and no serum‐derived endothelial BMP activity (Hodgson et al., 2021). In this study, both children had detectable levels of pBMP10, albeit lower than their parents. Having fully validated the BMP10 GFD ELISA, we show that BMP10 GFD levels were very low, but similar in the affected male child and unaffected father. Intriguingly, the BMP10 levels in the affected female child and unaffected mother were also similar and although higher than the male family members, they were lower than the female controls. This may suggest a genetic or epigenetic link between BMP10 levels in each child and their parent of the same sex. Also, the higher ratio of BMP10 GFD:pBMP10 in the female child suggests BMP10 processing to increase the availability of the GFD may be greater than in the other family members. Given these data, we speculate that the earlier disease onset and greater severity in the male homozygote child may be a consequence of BMP9 loss combined with low BMP10, whereas the impact of BMP9 loss in his sister may be mitigated by residual circulating BMP10.

We previously showed that loss of BMP9 and pBMP10 corresponded to the absence of serum‐derived BMP activity in the HMEC1‐BRE reporter line (Hodgson et al., 2021). Here, serum‐derived BRE‐luciferase activity was low, but evident in all four family members. As pBMP10 and/or BMP10 GFD levels were measurable, the activity in the homozygotes may represent BMP10 signaling, consistent with our previous immunoneutralization data (Hodgson et al., 2020). Atypical telangiectasias were evident in both homozygous patients (Q26X and E279X) in our previous report, both of whom lacked detectable plasma BMP9, pBMP10 and serum‐derived HMEC1‐BRE activity (Hodgson et al., 2021). Of note, the Q26X homozygous child was diagnosed with PAH at 3 years of age and telangiectasias at 10 years, but BMP9 and BMP10 levels were only measured at 10 years (Hodgson et al., 2021; Wang et al., 2016). In this study, we purposely assessed the family for telangiectasias and none were found. We suggest that the telangiectasias documented in homozygous *GDF2* variant carriers lacking BMP9 in our earlier study (Hodgson et al., 2021) may reflect the additional loss of circulating BMP10.

Current evidence suggests that pathogenic *GDF2* variants predispose to several vascular dysplasias (excepting non‐immune hydrops fetalis; Aukema et al., 2020), but additional genetic modifiers and/or somatic events are required to cause disease (Figure 2g; Table S1; Gallego et al., 2021). Potentially pathogenic heterozygous *GDF2* variants occur most frequently in adult PAH patients (74 cases) (Abou Hassan et al., 2018; Eyries et al., 2019; Hodgson et al., 2020; Wang et al., 2019; Zhu et al., 2019) compared to three members of the same family with hereditary hemorrhagic telangiectasia (HHT) (Balachandar et al., 2022) and three patients with a cutaneous malformation AVM phenotype (Wooderchak‐Donahue et al., 2013). Rare homozygous *GDF2* variants in children have been identified in PAH (Wang et al., 2016 and this study), possible PAH associated with HHT (Gallego et al., 2021), PAVM‐related disease (Hodgson et al., 2021; Liu et al., 2020) and non‐immune hydrops fetalis (NIHF) (Aukema et al., 2020). Also, Gallego et al. reported a child with IPAH with a missense mutation on the maternal allele and a large deletion encompassing *GDF2* and *BMP10* in the paternal chromosome 10, thus representing a compound heterozygote potentially exacerbated by the BMP10 deletion (Gallego et al., 2021). Critically, unaffected parents of homozygous pediatric cases are heterozygous for these variants (this study and Aukema et al., 2020; Gallego et al., 2021; Hodgson et al., 2021; Liu et al., 2020; Wang et al., 2016; Zhu et al., 2019), four of which (Q26X, R110W, R151X, S320C) have been identified in adult PAH cases (Table S1; Eyries et al., 2019; Hodgson et al., 2020; Wang et al., 2019; Zhu et al., 2019). In one case, a sibling homozygous for the Y354RfsTer15 was not affected (Liu et al., 2020). We have shown that unaffected heterozygous E279X (Hodgson et al., 2021) and S320C (this study) variant carriers have reduced plasma BMP9 levels. Thus, we hypothesize that pathogenic BMP9 variants predispose to disease and additional factors precipitate the emergence of disease pathologies, often in the pulmonary vasculature. At this stage, we cannot exclude the possibility that BMP9 mutations may impact on BMP10 processing or secretion in a mutation‐specific manner.

In conclusion, we report two pediatric siblings with PAH harboring a rare homozygous *GDF2* variant and confirm that loss of circulating BMP9 compounded by reduced BMP10 likely contributes to PAH onset. Unaffected heterozygous individuals with reduced BMP9 levels suggest that heterozygous deleterious *GDF2* variants predispose to disease but are not sufficiently causal.

## FUNDING INFORMATION

British Heart Foundation Programme Grant RG/19/3/34265.

## CONFLICT OF INTEREST

PDU is a founder of, and scientific advisor to Morphogen‐IX Ltd. NWM is a founder and CEO of Morphogen‐IX Ltd. PDU and NWM have published US (US10336800) and EU (EP3166628B1) patents entitled: “Therapeutic Use of Bone Morphogenetic Proteins.” All other authors declare no competing interests.

## Supporting information


Appendix S1:

## Data Availability

The data that support the findings of this study are available from the corresponding author upon reasonable request.
