# Supplementary material for: A rare homozygous missense GDF2 (BMP9) mutation causing PAH in siblings: Does BMP10 status contribute?
Source: Am J Med Genet A. 2022 Oct 19;191(1):228–33. doi: 10.1002/ajmg.a.62996 (PMC10092753; doi:10.1002/ajmg.a.62996)
Supplement: Supplementary file 1 — Appendix S1: [file AJMG-191-228-s001.docx]

**SUPPLEMENTAL INFORMATION**

**A rare homozygous missense *GDF2* (BMP9) mutation causing PAH in siblings: does BMP10 status contribute?**

Paul Upton^1^ | Susan Richards MN NP^2^ | Angela Bates, MD, FRCPC^2,3^ | Karen Y. Niederhoffer^4^ | Nicholas W. Morrell^1^ | Susan Christian^2^

^1^Department of Medicine, Heart and Lung Research Institute, University of Cambridge, Cambridge, UK

^2^Pediatric Pulmonary Hypertension Service, Stollery Children’s Hospital, Edmonton, Canada

^3^Department of Pediatrics, University of Alberta, Edmonton, Canada

^4^Department of Medical Genetics, University of Alberta Hospital, Edmonton, Canada

**CLINICAL DESCRIPTION**

The proband (E001), aged 11 years at the time of blood sampling, was originally diagnosed at 8 months with severe PAH in India. His initial diagnosis was made by his clinical presentation and echocardiogram findings that estimated a mPAP of 80 mmHg at 2.5 months of age. After assessment by a pediatric cardiologist, he was diagnosed with IPAH at 3 months of age and discharged home. He was stabilized on sildenafil, Lasix®, digoxin and Aldactazide®. Despite having overall improved energy, he continued to have tachypnea and recurrent pneumonias. After moving to Canada, he was seen at 9 months of age by the Stollery Pulmonary Hypertension team where he underwent a cardiac catheterization. He met criteria for IPAH and was initiated on dual therapy with the addition of bosentan after discharge. Follow up cardiac catheterizations showed significantly improved pressures and he has remained stable over the past several years. He is currently on oral PAH therapy with tadalafil (40 mg/day) and ambrisentan (10 mg/day). His other comorbidity includes chronic rhinitis with intermittent snoring. He currently has a WHO functional classification of 2 as he experiences shortness of breath with exercise. ^1^ He has remained stable throughout his early childhood years. At examination at the age of 11, heart rate was 93 bpm and systemic blood pressure was 105/60. A distance of 475m (51% predicted for age) was achieved during the 6-minute walk test, with good heart rate and blood pressure variability and an oxygen saturation drop from 95% to 91%. Assessment by 12-lead electrocardiogram (ECG) suggested a right axis deviation for age, but otherwise normal axis and intervals. In V1, the R-waves were slightly higher than the 98^th^ percentiles and R/S was greater than 1, suggesting right ventricular hypertrophy (RVH). Subsequent assessment by echocardiography demonstrated the right ventricle was mildly hypertrophied and dilated, with a normal systolic function. Right ventricular systolic pressure (RVSP) was estimated from tricuspid regurgitation as 49mmHg plus right atrial pressure, while systolic blood pressure was 101mmHg. Right ventricular flattening was evident in systole, consistent with the RVSP being approximately half systemic pressure. The left ventricle was normal in respect of size or systolic and diastolic functions. There was no evidence of right or left ventricular outflow tract obstructions and no pericardial effusion. His RV/LV end systolic ratio is 1.0, and eccentricity index is 1.1. A recent cardiac catheterization shows a mPAP of 33 mmHg, transpulmonary gradient (TPG) of 24 mmHg, a PCWP of 9 mmHg with a PVRI of 6.9 WU.m^2^. His cardiac index is 3.56 L/min/m^2^. He does not have reactivity with acute vasoreactivity testing.

The sister of the proband (E002), now aged 8 years at the time of blood sampling, was diagnosed at 4 years with IPAH. She had been monitored via the screening of siblings of all IPAH patients through our program. The mother noted a few months prior that the sister of the proband had less energy than normal. Her initial ECG was unremarkable and her echocardiogram at that time showed evidence of mildly increased RVSP estimated at half systemic, with a mPAP estimated by pulmonary insufficiency of 25 mmHg. She underwent a cardiac catheterization along with full assessment for etiology of PAH and was confirmed to have IPAH at 5 years old. Her cardiac catheterization showed a mPAP of 27 mmHg, TPG of 21 mmHg, PCWP of 6 and PVRI of 6.23 WU.m^2^. Her cardiac index was normal at 3.38 L/min/m^2^. She was started on therapy and is currently treated with tadalafil (30 mg/day) and ambrisentan (10 mg/day). Her only other significant past history was that of anemia, which was resolved at 3 years of age. She has a WHO functional classification of 1-2, as she intermittently experiences shortness of breath upon exercise. Upon examination at the age of 8, heart rate was 81bpm and systemic blood pressure was 117/54. A distance of 460m was achieved during the 6-minute walk test, with good heart rate and blood pressure variability and a saturation that declined from 94% to 93%, with a post-test Borg dyspnea score of 5. Assessment by 12-lead ECG suggested axis and intervals were all normal for age, with no indication of RVH in V1 and normal R-wave progression in V6. Her most recent echo shows well controlled disease with a round septal curvature suggesting less than half systemic RVSP. Her RV/LV end systolic ratio is 0.74 and eccentricity index of 1.1. There is not enough TR to estimate an RVSP.

Neither parent (father = 41years; mother = 38years) exhibited any health concerns. Echocardiographic assessment indicated the right and left ventricles were normal with ejection fractions >55%, tricuspid annular plane systolic excursion 2.1-2.2mm and normal RVSPs (father = 23mmHg; mother = 17mmHg. Although the mother and both children exhibited pin-point brown flat non-blanchable macules on the tongue, there is no recorded history of epistaxis, telangiectasias or arteriovenous malformations (AVMs) in this family.

Table S1: Summary of pathogenic BMP9 variants identified in heterozygotes and homozygotes with disease and also in unaffected individuals, implying reduced penetrance of *GDF2* mutations.

| BMP9 Variant | Disease status |
| --- | --- |
| p.Q26X | Heterozygous – unaffected adult^2^  Heterozygous – adult PAH^3^  Homozygous – pediatric PAH^2^ |
| p.R110W | Heterozygous – unaffected adult^4^  Heterozygous – adult PAH^5,6^  Homozygous – pediatric HHT with associated PAH^4^ |
| p.R151X | Heterozygous – unaffected adult^7^  Heterozygous – adult PAH^8^  Homozygous – NIHF^7^ |
| p.E279X | Heterozygous – unaffected^9^  Homozygous – Pulmonary AVMs^9^ |
| p.S320C | Heterozygous – unaffected adult (this study)  Heterozygous – adult PAH^5^  Homozygous – PAH (this study) |
| p.C428R | Heterozygous – adult PAH^6^  Heterozygous – pediatric HHT^10^  Heterozygous – adult HHT^10^ |
| p.Y354RfsTer15 | Homozygous – unaffected child^11^  Homozygous – pulmonary AVMs^11^ |

**REFERENCES**

1. Lammers, A.E., Adatia, I., Cerro, M.J., Diaz, G., Freudenthal, A.H., Freudenthal, F., Harikrishnan, S., Ivy, D., Lopes, A.A., Raj, J.U., et al. (2011). Functional classification of pulmonary hypertension in children: Report from the PVRI pediatric taskforce, Panama 2011. Pulmonary circulation *1*, 280-285. 10.4103/2045-8932.83445.

2. Wang, G., Fan, R., Ji, R., Zou, W., Penny, D.J., Varghese, N.P., and Fan, Y. (2016). Novel homozygous BMP9 nonsense mutation causes pulmonary arterial hypertension: a case report. BMC Pulm. Med. *16*, 17. 10.1186/s12890-016-0183-7.

3. Eyries, M., Coulet, F., Girerd, B., Montani, D., Humbert, M., Lacombe, P., Chinet, T., Gouya, L., Roume, J., Axford, M., et al. (2011). ACVRL1 germinal mosaic with two mutant alleles in hereditary hemorrhagic telangiectasia associated with pulmonary arterial hypertension. Clin.Genet.

4. Gallego, N., Cruz-Utrilla, A., Guillén, I., Bonora, A.M., Ochoa, N., Arias, P., Lapunzina, P., Escribano-Subias, P., Nevado, J., and Tenorio-Castaño, J. (2021). Expanding the Evidence of a Semi-Dominant Inheritance in GDF2 Associated with Pulmonary Arterial Hypertension. Cells *10*, 3178. 10.3390/cells10113178.

5. Hodgson, J., Swietlik, E.M., Salmon, R.M., Hadinnapola, C., Nikolic, I., Wharton, J., Guo, J., Liley, J., Haimel, M., Bleda, M., et al. (2020). Characterization of GDF2 Mutations and Levels of BMP9 and BMP10 in Pulmonary Arterial Hypertension. American journal of respiratory and critical care medicine *201*, 575-585. 10.1164/rccm.201906-1141OC.

6. Zhu, N., Pauciulo, M.W., Welch, C.L., Lutz, K.A., Coleman, A.W., Gonzaga-Jauregui, C., Wang, J., Grimes, J.M., Martin, L.J., He, H., et al. (2019). Novel risk genes and mechanisms implicated by exome sequencing of 2572 individuals with pulmonary arterial hypertension. Genome Medicine *11*, 69. 10.1186/s13073-019-0685-z.

7. Aukema, S.M., Ten Brinke, G.A., Timens, W., Vos, Y.J., Accord, R.E., Kraft, K.E., Santing, M.J., Morssink, L.P., Streefland, E., van Diemen, C.C., et al. (2020). A homozygous variant in growth and differentiation factor 2 (GDF2) may cause lymphatic dysplasia with hydrothorax and nonimmune hydrops fetalis. Am J Med Genet A *182A*, 2152-2160. 10.1002/ajmg.a.61743.

8. Eyries, M., Montani, D., Nadaud, S., Girerd, B., Levy, M., Bourdin, A., Tresorier, R., Chaouat, A., Cottin, V., Sanfiorenzo, C., et al. (2019). Widening the landscape of heritable pulmonary hypertension mutations in paediatric and adult cases. The European respiratory journal *53*, 1801371. 10.1183/13993003.01371-2018.

9. Hodgson, J., Ruiz-Llorente, L., McDonald, J., Quarrell, O., Ugonna, K., Bentham, J., Mason, R., Martin, J., Moore, D., Bergstrom, K., et al. (2021). Homozygous GDF2 nonsense mutations result in a loss of circulating BMP9 and BMP10 and are associated with either PAH or an "HHT-like" syndrome in children. Molecular genetics & genomic medicine *9*, e1685. 10.1002/mgg3.1685.

10. Balachandar, S., Graves, T.J., Shimonty, A., Kerr, K., Kilner, J., Xiao, S., Slade, R., Sroya, M., Alikian, M., Curetean, E., et al. (2022). Identification and validation of a novel pathogenic variant in GDF2 (BMP9) responsible for hereditary hemorrhagic telangiectasia and pulmonary arteriovenous malformations. Am J Med Genet A *188*, 959-964. 10.1002/ajmg.a.62584.

11. Liu, J., Yang, J., Tang, X., Li, H., Shen, Y., Gu, W., and Zhao, S. (2020). Homozygous GDF2-Related Hereditary Hemorrhagic Telangiectasia in a Chinese Family. Pediatrics *146*, e20191970. 10.1542/peds.2019-1970.
